# Cell Cycle Inhibition To Treat Sleeping Sickness

**DOI:** 10.1128/mBio.01427-17

**Published:** 2017-09-19

**Authors:** Conrad L. Epting, Brian T. Emmer, Nga Y. Du, Joann M. Taylor, Ming Y. Makanji, Cheryl L. Olson, David M. Engman

**Affiliations:** a Department of Pediatrics, Northwestern University, Chicago, Illinois, USA; b Department of Pathology, Northwestern University, Chicago, Illinois, USA; c Department of Microbiology-Immunology, Northwestern University, Chicago, Illinois, USA; d Department of Pathology and Laboratory Medicine, Cedars Sinai Medical Center, Los Angeles, California, USA; Albert Einstein College of Medicine

**Keywords:** African sleeping sickness, *Trypanosoma brucei*, hydroxyurea, ribonucleotide reductase

## Abstract

African trypanosomiasis is caused by infection with the protozoan parasite *Trypanosoma brucei*. During infection, this pathogen divides rapidly to high density in the bloodstream of its mammalian host in a manner similar to that of leukemia. Like all eukaryotes, *T. brucei* has a cell cycle involving the *de novo* synthesis of DNA regulated by ribonucleotide reductase (RNR), which catalyzes the conversion of ribonucleotides into their deoxy form. As an essential enzyme for the cell cycle, RNR is a common target for cancer chemotherapy. We hypothesized that inhibition of RNR by genetic or pharmacological means would impair parasite growth *in vitro* and prolong the survival of infected animals. Our results demonstrate that RNR inhibition is highly effective in suppressing parasite growth both *in vitro* and *in vivo*. These results support drug discovery efforts targeting the cell cycle, not only for African trypanosomiasis but possibly also for other infections by eukaryotic pathogens.

## IMPORTANCE

The development of drugs to treat infections with eukaryotic pathogens is challenging because many key virulence factors have closely related homologues in humans. Drug toxicity greatly limits these development efforts. For pathogens that replicate at a high rate, especially in the blood, an alternative approach is to target the cell cycle directly, much as is done to treat some hematologic malignancies. The results presented here indicate that targeting the cell cycle via inhibition of ribonucleotide reductase is effective at killing trypanosomes and prolonging the survival of infected animals.

## INTRODUCTION

The protozoan *Trypanosoma brucei* is the causative agent of human African trypanosomiasis (HAT), also known as sleeping sickness (1). HAT is an endemic disorder of poverty in vast regions of sub-Saharan Africa afflicting thousands of individuals annually (2). Metacyclic trypomastigotes in the salivary gland of the tsetse fly vector are transmitted to humans and other mammals when the fly takes a blood meal and initially invade local lymphatics, differentiate into bloodstream trypomastigotes, and then divide in the bloodstream to high levels. Although adaptive immunity against *T. brucei* surface coat antigens is effective at clearing the infection, the parasite persists in the host by sequentially shedding and changing its coat of variable surface glycoproteins (VSG) in a process called antigenic variation (3, 4). Subsequent waves of parasitemia eventually disrupt the blood-brain barrier, causing neurologic disability and death if unrecognized and untreated (5–7). Two subspecies of *T. brucei* cause human disease, *T. b. gambiense* and *T. b. rhodesiense*, while most investigators use the animal-adapted substrain *T. b. brucei* for research because it fails to infect humans because of the lytic action of apolipoproteins (8). During infection, parasites reach a high cell density in the bloodstream through rapid cell proliferation, induction of host immunosuppression through altered lymphocyte and macrophage differentiation, and evasion of host immunity via antigenic variation (3, 4). The few available approved treatments are relatively toxic, have led to the emergence of drug resistance, and are difficult to deliver, thus limiting their effectiveness in areas where HAT is endemic that may have limited access to advanced medical care and intravenous (i.v.) medications (9–11). Given the complexity of delivering care in these areas, the development of easy-to-administer and inexpensive trypanocidal therapies is needed.

There have been important advances in antitrypanosome drug development. Nifurtimox-eflornithine combination therapy (NECT) has proven very successful against later-stage infection (12), and there is ongoing development of drugs targeting sterols, histone deacetylation, and proteasomes (9, 13–15). These treatments exploit unique or vulnerable aspects of trypanosome biology, and further study will ideally yield highly selective agents with minimal host toxicity. Unfortunately, as with many neglected tropical diseases, the advancement of novel treatments for HAT is often slowed as pharmaceutical companies struggle to recover research-and-development costs. This has led some to explore drug repurposing—utilizing existing drugs with proven safety profiles and preexisting clinical experience—for the treatment of other diseases (16).

With this in mind, we considered alternative approaches to treatment and were struck by three aspects of HAT—the eukaryotic nature of trypanosomes, their bloodstream niche, and their high growth rate (17). These are shared properties of cancers of the blood, such as leukemia, and we thought to approach the treatment of *T. brucei* infection in a similar fashion to model a nonspecific chemotherapeutic approach targeting a rapidly dividing eukaryotic cell. Congruent with this approach, new candidate anticancer compounds often have significant activity against trypanosomes *in vitro* (18), and a similar approach has already been applied by using antimetabolites directed against trypanosome pyruvate kinase (19). Cancer treatment routinely employs nonspecific cell cycle inhibitors, the actions of which exploit the relatively high rate of division of malignant cells, thus differentially impacting neoplastic cells over host tissues.

Ribonucleotide reductase (RNR), a conserved enzyme complex essential for cell proliferation (20), is a common target for antineoplastic agents (21, 22). Hydroxyurea, an orally available antineoplastic agent, inhibits RNR with a well-characterized safety profile and remains in use even today for the treatment of certain myeloproliferative disorders (23, 24). Hydroxyurea has been used experimentally for cell cycle synchronization of *T. brucei* (25), but this is the first report exploring the use of hydroxyurea for the *in vivo* treatment of African trypanosomiasis in a murine model.

## RESULTS

### Inhibition of RNR attenuates parasite growth.

**FIG 1:**
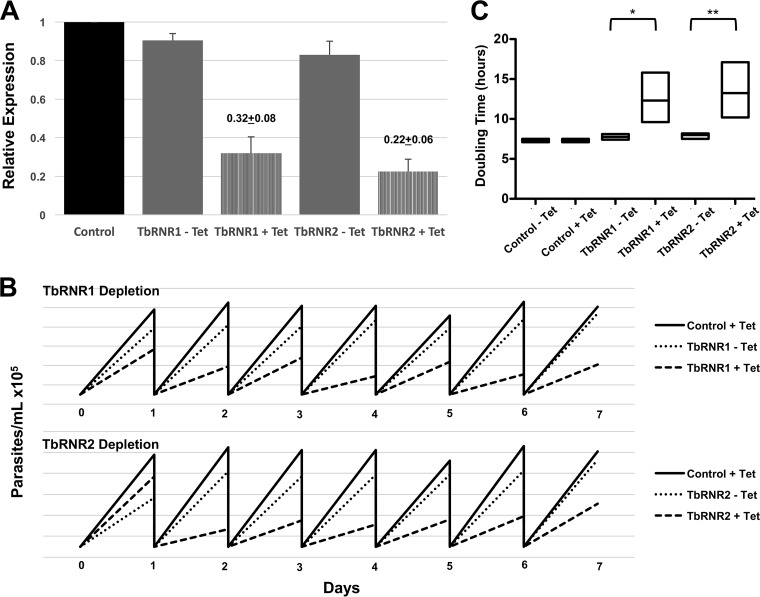
RNR depletion attenuates parasite growth and prolongs the parasite doubling time *in vitro*. Bloodstream form parasites expressing a tetracycline-inducible RNAi construct against TbRNR1 and TbRNR2 were induced in the presence of tetracycline or the vehicle on day 0 and subcultured to keep cultures in log-phase growth between 1 × 10^5^ and 2.5 × 10^6^ parasites/ml. (A) Knockdown in the presence of tetracycline (+Tet) was confirmed by qPCR after 48 h. Relative mRNA expression was normalized against no-tetracycline (–Tet) controls based on cycle time to detection, revealing a 68% reduction of TbRNR1 mRNA and a 78% reduction of TbRNR2. (B) Rate of parasite expansion plotting cell number against time during daily subculture back to 1 × 10^5^/ml to maintain log-phase growth. The addition of tetracycline to induce RNAi against TbRNR1 or TbRNR2 suppressed parasite growth significantly compared to that of wild-type or no-tetracycline controls. The average results from three independent experiments cultured in triplicate are shown with the SEM. The intermediate phenotype in the no-tetracycline (−Tet) group may represent leakage of the inducible construct and the sensitivity to RNR-mediated cell cycle suppression. (C) Doubling time prolongation reflected in floating box plots (min/max, line at mean) demonstrating that RNAi against TbRNR1 or TbRNR2 significantly prolonged the mean doubling time over 7 days compared to that of uninduced (−Tet) controls with an unpaired *t* test to compare the presence and absence of tetracycline. TbRNR1, 7.8 ± 0.1 h with tetracycline versus 12.3 ± 0.8 h without tetracycline (*, *P* = 0.0082); TbRNR2, 8 ± 0.1 h with tetracycline versus 13.3 ± 0.8 h without tetracycline (**, *P* = 0.0039).

To investigate RNR inhibition as a potential therapeutic target for HAT, we first identified two RNR enzyme subunits in the *T. brucei* genome resource TritrypDB (26)—TbRNR1 (large chain [GenBank accession number U80910, TritrypDB accession numbers Tb427tmp.02.5720 and Tb927.11.7840]) and TbRNR2 (small chain [GenBank accession number U80911, TritrypDB accession numbers Tb427tmp.11.0004 and Tb927.11.12780]). We generated *T. brucei* cell lines containing tetracycline-inducible RNA interference (RNAi) constructs targeting TbRNR1 and TbRNR2. Induction of RNAi reduced the levels of the TbRNR1 and TbRNR2 mRNAs significantly (Fig. 1A, TbRNR1, 0.32 ± 0.08; TbRNR2, 0.22 ± 0.06; *n* = 2). Knockdown of TbRNR1 or TbRNR2 caused a growth deficit (Fig. 1B) and significant prolongation of the mean doubling time (Fig. 1C), with RNR1 depletion increasing the doubling time from 7.8 ± 0.1 to 12.3 ± 0.8 h (*P* = 0.0082) and RNR2 depletion increasing the doubling time from 8 ± 0.1 to 13.3 ± 0.8 h (*P* = 0.0039).

### Hydroxyurea is more toxic to *T. brucei* than to human cells.

**FIG 2:**
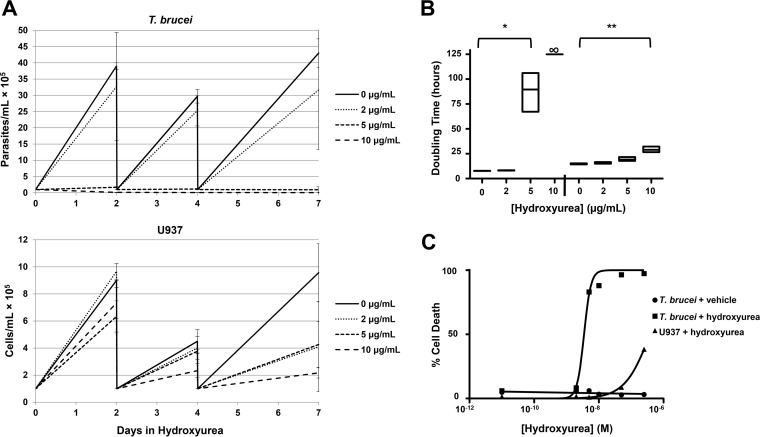
Hydroxyurea is more toxic to *T. brucei* than to mammalian cells *in vitro*. *T. brucei* parasites are more sensitive to growth inhibition by hydroxyurea than were mammalian U937 (human lymphoma) cells. (A) Growth rates of *T. brucei* (top) and U937 cells (bottom) after periodic subculture in the presence of increasing concentrations of hydroxyurea (0 to 10 μg/ml) for 7 days. Growth arrest at >5 μg/ml hydroxyurea was observed in the *T. brucei* cultures. The average results of three independent experiments done in triplicate are shown with the SEM. (B) Mean doubling time (floating box plots to min/max, line at mean) demonstrate that the doubling time was significantly prolonged for *T. brucei* at 5 μg/ml (*, *P* < 0.001 versus the control) and for U937 at 10 μg/ml (**, *P* = 0.0011 versus the control), yielding an increase in the doubling time at 5 μg/ml of 11.5-fold for *T. brucei* and 1.3-fold for U937, supporting the idea that *T. brucei* is more sensitive to hydroxyurea than mammalian cells are. (∞ denotes that *T. brucei* cultured in 10 μg/ml hydroxyurea was functionally nonproliferative; one-way ANOVA was used to test significance) (C) To determine toxicity, cells in log-phase growth were inoculated at 1 × 10^5^/ml and exposed to hydroxyurea over a 3-log range (0 to 500 μg/ml) for 48 h. Cell numbers and cell death were quantified by using exclusion of either propidium iodide (parasites) or trypan blue (U937). Cell losses were additive when remaining cells were less numerous than those in the inoculum. The LD_50_ for *T. b. brucei* bloodstream forms was significantly lower than for U397 cells (3.4 versus 385 μg/ml; *P* < 0.0001, *n* = 3). The result of vehicle treatment of *T. brucei* is also shown.

**FIG 3:**
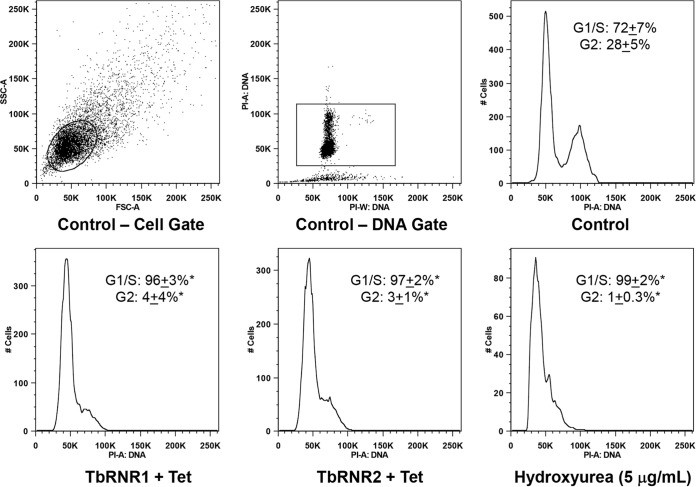
RNR depletion and hydroxyurea treatment result in accumulation of cells in G_1_/S phase. Cells were cultured in the presence or absence of tetracycline (Tet), to induce RNAi against TbRNR1 or TbRNR2, or hydroxyurea (5 μg/ml) for 2 days. DNA was stained with propidium iodide and analyzed by flow cytometry to obtain univariate DNA histograms by gating on cells and DNA content. Representative histograms are shown. The frequencies of G_1_, S, and G_2_ cells were analyzed by ModFit. The percentages of cells in G_1_/S and G_2_ are shown within each histogram, demonstrating the accumulation of cells in G_1_/S after depletion of TbRNR1 or TbRNR2 or treatment with hydroxyurea. *, significantly different from the control population by unpaired *t* test comparing the two groups (i.e*.*, control versus treated G_1_/S cells), *P* < 0.05, *n* = 4.

Next we determined the relative effectiveness (as measured by prolongation of the doubling time) and toxicity (as measured by the 50% lethal dose [LD_50_]) of hydroxyurea for both *T. brucei* bloodstream trypomastigotes and a human cell line. The lymphoma cell line U937 (ATCC 1593.2) was selected as a representative cell line with which to model host toxicity, since bone marrow suppression is a dose-limiting side effect of hydroxyurea treatment in humans. Increasing concentrations of hydroxyurea effectively suppressed the growth of *T. brucei*, with a doubling time (mean number of hours ± the standard error of the mean [SEM]) of 7.72 ± 0.06 at 0 μg/ml, 8.2 ± 0.1 at 2 μg/ml, and 89.5 ± 11.6 at 5 μg/ml (*P* < 0.001). Treatment with 10 μg/ml was toxic, and cells stopped dividing and died (arbitrarily graphed at ∞ = 125 h). U937 cells were far more resistant to hydroxyurea, doubling in 14.7 ± 0.3 (mean ± SEM) h at 0 μg/ml, 15.94 ± 0.5 h at 2 μg/ml, 19.2 ± 1.2 h at 5 μg/ml (no statistically significant difference), and 29 ± 1.7 h at 10 μg/ml (*P* = 0.0011) (Fig. 2B). When normalized to the differing rates of cell division (7.7 h for *T. brucei* versus 14.7 h for U937 cells), treatment with hydroxyurea at 5 μg/ml resulted in an 11.5-fold division impairment of *T. brucei* and a 1.3-fold division impairment of U937 cells. Next, we determined the relative cellular toxicity by the *in vitro* LD_50_ over a 3-log range (0 to 500 μg/ml = 10^−11^ to 10^−6.5^ M) for hydroxyurea treatment against *T. brucei* and U937 cells by exclusion of propidium iodide (for *T. brucei*) or trypan blue (for U937), respectively. Cell losses were considered additive when the number of cells remaining after treatment was less than that in the inoculum. The LD_50_ was 3.4 μg/ml for *T. brucei* versus 385 μg/ml for U937 (*P* < 0.0001, Fig. 2C), indicating a wide therapeutic index for hydroxyurea treatment. We suspected that inhibition of RNR, by either genetic (RNAi) or pharmacological (hydroxyurea) means, should result in accumulation of cells in the G_1_/S stage of the cell cycle, with fewer cells in G_2_ than in an asynchronous dividing population. Flow cytometric analysis of DNA content confirmed that RNR inhibition by RNAi or treatment with hydroxyurea causes cell cycle arrest in G_1_/S (Fig. 3).

### Hydroxyurea suppresses parasitemia and prolongs animal survival *in vivo*.

**FIG 4:**
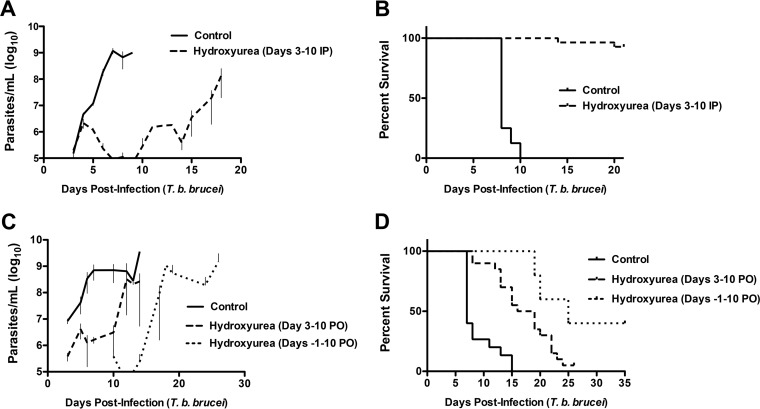
Hydroxyurea suppresses parasitemia and prolongs animal survival *in vivo* following infection with *T. b. brucei*. (A and B) To determine if hydroxyurea would suppress parasitemia, 40 BALB/c mice were infected with *T. b. brucei* and treated with hydroxyurea (500 mg/kg/day) or PBS (control) delivered i.p. for 7 days and observed for 21 days. (A) Hydroxyurea-treated mice demonstrated significantly attenuated parasitemia curves (*P* < 0.0001 versus the control). (B) Kaplan-Meier survival curves demonstrating significantly prolonged median survival with hydroxyurea treatment (control, 8 days; treatment, 20.3 days; *P* < 0.0001). (C and D) To determine if oral (PO) hydroxyurea therapy is efficacious, 65 mice were infected on day 0 and observed for 35 days. PBS (control) or hydroxyurea at 500 mg/kg/day was administered in the water supply as prophylaxis (days −1 to +10) or as treatment (days 3 to 10) following infection. (C) Parasitemia curves demonstrate that prophylaxis and treatment were significantly different from the control (*P* = 0.04046 and 0.01686, respectively). (D) Kaplan-Meier survival curves recorded over 35 days demonstrate a significant prolongation of the median survival time for hydroxyurea prophylaxis (control [PBS], 7 days; hydroxyurea prophylaxis, 25 days; *P* < 0.0001) and treatment (control, 7 days; hydroxyurea, 17.5 days; *P* = 0.00028). Mice surviving to the end of the study (40% of the hydroxyurea prophylaxis group) were screened by qPCR and confirmed to be negative (<100 *T. brucei* copies/ml), supporting the increased effectiveness of prophylactic hydroxyurea exposure (PCR data are not shown). Determination of the statistical significance of differences in survival time was performed by log rank (Mantel-Cox) analysis.

When infected with 1,000 *T. brucei* bloodstream trypomastigotes, mice generally develop an unremitting infection leading to high parasitemia (>10^9^/ml) and death within 1 to 2 weeks. To determine the effect of hydroxyurea therapy in an animal model of sleeping sickness, we treated *T. brucei*-infected BALB/c mice with hydroxyurea. Mice were infected with 1,000 *T. brucei* bloodstream trypomastigotes, and 3 days later, when infection was established and parasitemia was generally ≥10^5^/ml, hydroxyurea was administered by daily intraperitoneal (i.p.) injection (500 mg/kg/day) for 7 days. Hydroxyurea-treated mice demonstrated significantly reduced parasitemia (Fig. 4A) and prolonged survival (Fig. 4B) over a 21-day monitoring period (mean parasitemia ± SEM is shown; average survival time: control, 8 days; hydroxyurea, 20.3 days [*P* < 0.001, *n* = 40 mice]). Cessation of hydroxyurea therapy following a week of therapy (at day 10) resulted in parasite reemergence (Fig. 4A).

We next tested whether hydroxyurea would be effective when given orally and whether pretreatment (prophylaxis) would better suppress parasite replication and prolong animal survival. We administered hydroxyurea or the vehicle to mice (*n* = 65) in their drinking water (500 mg/kg/day) as treatment (from day 3 to day 10 postinfection) or as prophylaxis (from day −1 to day 10 postinfection). Mice were infected with 10^4^ *T. brucei* bloodstream trypomastigotes and monitored for 35 days. As expected, both strategies were effective in suppressing parasitemia (prophylaxis versus the control, *P* = 0.04046; treatment versus the control, *P* = 0.01686; Fig. 4C). Animal survival was similarly prolonged either with prophylaxis (prophylaxis versus the control, 25 versus 7 days, *P* < 0.0001; Fig. 4D) or with treatment (treatment versus the control, 17.5 versus 7 days, *P* = 0.00028). In addition, ~40% of the animals in the prophylaxis arm never displayed any parasites in their blood, and quantitative PCR (qPCR) testing for *T. brucei* DNA was negative to a sensitivity of <100 copies/ml of blood, indicating that both oral treatment and prophylaxis were suppressive and, in some cases, potentially curative.

### Hydroxyurea suppresses parasitemia and prolongs animal survival following infection with the human pathogen *T. b. rhodesiense*.

**FIG 5:**
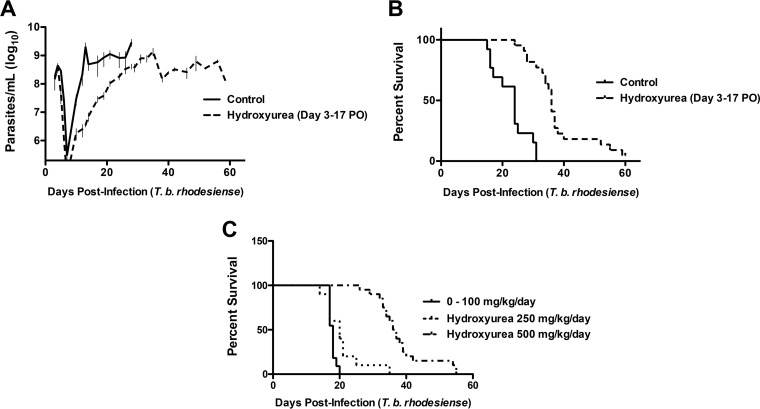
Hydroxyurea suppresses parasitemia and prolongs animal survival *in vivo* following infection with human-pathogenic *T. b. rhodesiense*. Seventy BALB/c mice were infected with *T. b. rhodesiense* on day 0 and treated with hydroxyurea at 500 mg/kg/day administered PO starting on day 3 following infection for 14 days. (A) Parasitemia curves reveal that treated mice had significantly lower parasitemia (*P* < 0.0001 versus the control) during treatment. (B) The median survival time of control mice over a 60-day observation period was 24 days versus 36 days for those treated with hydroxyurea (*P* < 0.0001). No animals survived to the end of the observation period once taken off hydroxyurea therapy. (C) Dose-response survival curves for hydroxyurea demonstrate that ≥250 mg/kg/day is necessary for a survival benefit following infection with *T. b. rhodesiense*. There was no statistically significant difference in survival time between control animals and those treated with hydroxyurea at 0 to 100 mg/kg/day, and visually irrelevant differences thus are represented by the control curve only as 0 to 100 mg/kg/day. Survival was significantly different between the control and treatment at 250 to 500 mg/kg/day, with a median survival time of 18 days for control mice versus 20 days for those treated at 250 mg/kg (*P* = 0.0327) and 36.5 days for those treated at 500 mg/kg/day (*P* < 0.0001). Determination of the statistical significance of differences in survival time was performed by log rank (Mantel-Cox) analysis.

*T. b. brucei* is nonpathogenic to humans because it is sensitive to lysis by serum apolipoproteins (8); thus, we sought to extend our observation to infection with a relevant human pathogen, *T. b. rhodesiense* (gift of J. Mansfield, University of Wisconsin). During *T. b. rhodesiense* infection, the host adaptive immune response is more effective than during infection with *T. b. brucei*, which extends animal survival. In the BALB/c mouse, parasitemia rises and falls every 6 to 8 days because of antigenic variation, successive rounds of clonal expansion, development of humoral immunity to the VSG surface coat, parasite clearance, switching of VSG gene expression, and reemergence of the new variant (27), although ultimately the mice succumb to infection (28). We reasoned that a more effective immune response might improve the effectiveness of hydroxyurea therapy, leading to parasite clearance rather than only suppression. To test this, we subjected 70 mice to an extended course of treatment (14 days, from day 3 to day 17 postinfection) and extended our observation period to 60 days. Similar to what we observed with *T. b. brucei*, hydroxyurea treatment reduced the replication rate during the first relapse and delayed death (Fig. 5A and B). However, all infected animals eventually succumbed to infection.

Reasoning that the off-target immunosuppressive effects of hydroxyurea therapy may limit the curative effects of treatment, we sought to determine the minimum effective dose of hydroxyurea necessary to prolong animal survival. We infected 35 mice with *T. b. rhodesiense*, treating them orally for 14 days with different doses of hydroxyurea ranging from 0 to 500 mg/kg/day. No difference was observed in mice treated with hydroxyurea at concentrations of 0 to 100 mg/kg/day (Fig. 5C) (median survival times: control, 18 days; treated, 17.2 days; *P* = 0.68). The median survival time of mice treated with 250 mg/kg/day (20 days versus 18 days for the control; *P* = 0.0327) or 500 mg/kg/day 36.5 days versus 18 days for the control; *P* < 0.0001) was significantly greater. We examined the bone marrow-suppressive effects of hydroxyurea therapy by measuring the peripheral white blood cell count as a surrogate for marrow suppression during infection with *T. b. rhodesiense*. Differences between groups did not reach statistical significance, although they did trend toward an expected immunosuppressive impact of treatment (mean white blood cell [WBC] counts at day 17: uninfected/no hydroxyurea, 1.4 × 10^7^/ml; infected/no hydroxyurea, 2.4 × 10^7^/ml; uninfected/hydroxyurea at 500 mg/kg/day, 0.8 × 10^7^/ml).

## DISCUSSION

Despite recent advances in drug discovery for HAT, current therapies are suboptimal. The four monotherapies, including pentamidine, suramin, melarsoprol, and eflornithine, suffer from limited effectiveness against a particular stage of infection, toxicity, difficulty in administration, or high cost (29). Combination therapies, for example, NECT, show promise, and it is hoped that replacing eflornithine, which must be administered by injection, with another oral agent like nifurtimox will advance therapy significantly. Resistance to eflornithine is also an emerging problem (30). Hydroxyurea has many characteristics of an ideal pharmaceutical—it is orally available, thermostable, and off patent and has a long history of clinical utility and safety. Thus, it is well suited for the treatment of *T. brucei* infection if proven effective and safe for the treatment of infected humans or livestock.

In this work, we discovered that inhibition of RNR by either RNAi or hydroxyurea suppresses parasite growth (Fig. 1 and 2) via cell cycle arrest at G_1_/S (Fig. 3) and prolongs animal survival following infection with *T. brucei* (Fig. 4 and 5). The LD_50_ of hydroxyurea after 48 h for *T. brucei* bloodstream cells is 3.4 μg/ml, which is comparable to the 4.2 μg/ml of eflornithine (31). Hydroxyurea monotherapy inhibits parasite replication during infection; however, parasites quickly rebound when therapy is withdrawn (Fig. 4). Hydroxyurea prophylaxis potentially can prevent infection, although this would never be indicated for a population in which the prevalence of *T. brucei* infection is relatively low. It should be noted that *T. brucei* infection causes immunosuppression (32, 33), and perhaps this may explain why hydroxyurea treatment did not cure infection in our experiments. Since *T. b. brucei* infection is normally 100% lethal in these mice, hydroxyurea treatment is clearly beneficial. Further, we never titrated the dose of hydroxyurea to the point of clear host toxicity (i.e., the LD_50_), so it is possible that this drug would be more effective in larger doses. While hydroxyurea might very well be effective as monotherapy during high parasitemia or if used in larger doses, our work suggests that it may be better as an early component of combination therapy, for example, with nifurtimox.

It is clear that therapies directed against the cell cycle for rapidly dividing eukaryotic pathogens are likely to be efficacious. Thinking about *T. brucei* and other eukaryotic pathogens, like *Plasmodium*, as “tumors of the blood” seems a reasonable way to approach drug discovery. Indeed, the idea of targeting the parasite cell cycle for chemotherapy was discussed long ago by Hammarton et al. (34), and hydroxyurea kills *Leishmania* amastigotes *in vitro* as well (35). Hydroxyurea therapy is used to prevent a sickle cell crisis in patients with sickle cell anemia, primarily by inducing the production of fetal hemoglobin, which prevents erythrocytes from sickling (36). There was concern that hydroxyurea prophylaxis for sickle cell anemia might alter the incidence of severe malaria for two reasons. First, the geographic distributions of sickle cell anemia and malaria largely overlap in sub-Saharan Africa. Second, hydroxyurea upregulates ICAM-1, a red cell receptor for *Plasmodium*. As we might have predicted, a study by Pino et al. on the effects of hydroxyurea on *Plasmodium* found the drug to actually inhibit parasite growth (37) and a phase III trial is under way to test the impact of hydroxyurea therapy administered for sickle cell prophylaxis on the incidence of malaria (38), further supporting the relevance of our work.

## MATERIALS AND METHODS

### Trypanosomes and mammalian cells.

*T. b. brucei* bloodstream form “single-marker” strain (39) parasites were cultured at 37°C with 5% CO_2_ in HMI-9 medium (40) supplemented with 10% fetal bovine serum (FBS), 10% serum plus medium complement (SAFC Biosciences), 100 U/ml penicillin/streptomycin, and 15 μg/ml G418. Transfectants were cultured under continuous drug selection with 2.5 μg/ml phleomycin (InvivoGen, San Diego, CA). Cultures were periodically subcultured to maintain log-phase growth between 1 × 10^5^/ml and 2.5 × 10^6^/ml. Transgenic parasites expressing the RNAi constructs were induced by the addition of 1 µg/ml tetracycline or the vehicle to the culture medium. *T. b. rhodesiense* -infected blood (the kind gift of J. Mansfield, University of Wisconsin) was propagated in mice (see below). U937 human lymphoma cells (ATCC, the kind gift of L. C. Platanias, Northwestern University) were passaged in RPMI 1640 supplemented with 10% FBS. Live/dead scoring was achieved by trypan blue exclusion for mammalian cells or propidium iodide exclusion for trypanosomes (which uniformly stain with trypan) observed through a Texas Red filter set on an inverted phase-contrast microscope.

### Animal experiments.

Infection of 8- to 12-week-old female BALB/c mice (Jackson Laboratories) was initiated by the i.v. or i.p. injection of 10^3^ to 10^4^ bloodstream form *T. b. brucei* parasites (respectively) or 10^5^ bloodstream form *T. b. rhodesiense* parasites suspended in 100 μl of PBS plus 1% glucose at 37°C. Mice intended as amplification hosts for *T. b. rhodesiense* were treated for 24 h prior to and 5 days following infection with immunosuppressive cyclophosphamide at 300 mg/kg i.p. daily to prevent VSG-clone switching, harvested between days 5 and 6 postinfection, and frozen aliquots were stored in 14% glycerol at −80°C until use (41). Forty animals were used in two experiments to establish the effectiveness of i.p. hydroxyurea, and 65 animals were used in three experiments supporting oral hydroxyurea therapy. We employed 105 animals in two experiments to support the effectiveness of hydroxyurea against *T. b. rhodesiense*. Serial parasitemia and WBC counts were measured by diluting 2.5 μl of blood obtained by tail snip every 1 to 3 days in a red blood cell lysis buffer and manual counting on a hemocytometer under phase-contrast microscopy at ×20 to ×40 magnification. Animal health was monitored frequently during infection for grooming, fighting, or weight loss. Animals receiving hydroxyurea alone experienced a 10 to 15% weight loss while on therapy, with recovery following termination of therapy, and there were no unexpected deaths during hydroxyurea therapy alone. Animals were euthanized if they were clinically moribund, demonstrated neurologic disability (seizures, hind limb paralysis, weakness), or had a parasite count of ≥10^9^/ml. All experimental procedures were approved by the Northwestern University IACUC and carried out in the Center for Comparative Medicine at Northwestern University.

### Hydroxyurea.

Hydroxyurea (Sigma) was solubilized in water, filter sterilized, and stored at 4°C. Cultures of parasites or cells were treated with 0 to 500 μg/ml hydroxyurea for 7 days as indicated, and fresh drug was added at the time of buffer exchange or medium dilution, generally every 1 to 2 days. Animals were treated daily i.p. with hydroxyurea freshly diluted in 100 μl of PBS or orally with hydroxyurea freshly diluted in 2.5% sucrose at 0 to 500 mg/kg/day for 8 to 14 days as indicated. Treatment was not initiated until day 3, when the average parasite count was ≥10^5^/ml for *T. b. brucei* or >10^6^ to 10^7^/ml for *T. b. rhodesiense* to ensure the establishment of infection. Intended oral dosing was calculated on the basis of the average daily water consumption per mouse and is reported as the intended dose. The actual dosage was monitored by weighing the cage water bottles; generally, the actual average dose received was 50 to 60% of the intended dose based on the number of animals remaining, although further precision on a per-mouse basis was not determined.

### RNR RNAi.

Sequences corresponding to the genes for TbRNR1 and TbRNR2 were identified in the *T. brucei* genome database (http://www.tritrypdb.org) (26), revealing two genes encoding the two RNR enzyme subunits (TbRNR1, large chain [GenBank accession number U80910]; TbRNR2, small chain [GenBank accession number U80911.1]). Optimal RNAi target sequences were identified with RNAit software (42) to minimize similarity to off-target genes. Oligonucleotides (Integrated DNA Technologies, Inc., Coralville, IA) were designed to amplify the target region and provide flanking HindIII and XhoI sites. PCR products were produced by *Taq* DNA polymerase (Invitrogen, Carlsbad, CA) amplification of genomic DNA, purified as previously described (43), and ligated into pCR2.1-TOPO (Invitrogen) prior to subcloning into the pZJM vector (44). To generate transgenic parasites, 50 μg of NotI-linearized pZJM-RNR1 or pZJM-RNR2 in 100 μl of sterile water was mixed with 2.25 × 10^7^ parasites in 450 μl of electroporation medium (120 mM KCl_2_, 0.15 mM CaCl_2_, 9.2 mM K_2_HPO_4_, 25 mM HEPES, 2 mM EDTA, 4.75 mM MgCl_2_, 69 mM sucrose, pH 7.6). The DNA-cell suspension was transferred to a cuvette with a 0.4-cm gap, electroporated twice (Gene Pulser electroporator; Bio-Rad, Hercules, CA) at 1.4 kV and 25 μF, and collected in 5 ml of fresh SDM-79 (Medium 2831; ATCC, Manassas, VA). Phleomycin selection was initiated at 16 h posttransfection.

### Real-time qPCR.

Total RNA was isolated from 1 × 10^8^ *T. brucei* with Trizol (Invitrogen, CA), and genomic DNA was cleaved with DNase I (Invitrogen). cDNA was synthesized from 3 μg of total RNA with oligo(dT) primers from the SuperScript III first-strand synthesis system for reverse transcription-PCR (Invitrogen). Real-time qPCR was performed on an iCycler system with iQ SYBR green Supermix (Bio-Rad Laboratories, CA) in eight-well strip tubes. The gene for α-tubulin was used as a reference to obtain a relative fold change for target samples by the comparative ΔΔ*C*_*T*_ method. Primers were designed with the Integrated DNA Technologies Primer Quest tool. The primers used were as follows (5′ to 3′): α-tubulin forward, GTGACGTTGTGCCAAAGGATGTGA; α-tubulin reverse, AGACCAGTCCACGAACTGAATCGT; RNR1 forward, TCATGGGACTTGGACGCTTATGGA; RNR1 reverse, GGGCCTGAATAGTTCTTACGCCA; RNR2 forward, ATGGGCGATTGAATGGATTGGCAG; RNR2 reverse, AATATGCCTTCGACAGCCGCAAAC. A 78- to 129-bp region outside the RNAi target sequence was amplified.

### DNA analysis by flow cytometry.

Parasites were harvested from culture after 48 h of RNAi induction with tetracycline and/or treatment with 5 μg/ml hydroxyurea, centrifuged at 2,000 × *g*, washed in PBS, fixed, and made permeable in 25% ethanol overnight at 4°C. Propidium iodide at 40 μg/ml was added, and at least 5,000 events were collected on a BD LSRII cytometer with standard filter sets. DNA histograms were analyzed with ModFit software for calculation of G_1_/S and G_2_ populations. No adjustment was made to account for kinetoplast versus nuclear DNA content in the ModFit algorithms.

### Analysis and statistics.

Cell numbers *in vitro* and *in vivo* were scored directly via microscopy. The parasite doubling time was calculated as 1/(log[final concentration] − log[initial concentration] × 3.32/number of hours between intervals). Each animal death was recorded as the actual day of death, the adjusted day of death, or the day of sacrifice for clinical signs. Well-appearing animals sacrificed empirically for humane reasons, on the basis of our extensive observed natural history of infection, had their day of death adjusted to apply a consistent metric to enable group analysis. Animals receiving euthanasia for a humane endpoint on the basis of high parasitemia were assigned a date of death for survival analysis as follows: ≥10^8^/ml (death in 48 h), the day of euthanasia was death on day +2; ≥10^9^/ml (death in 24 h), the day of euthanasia was death on day +1; ≥10^10^/ml, the day of euthanasia was the day of death. Oral hydroxyurea dosage data are reported as the intention to treat, as the actual consumption tended to be 50 to 70% of the intended dose. Excel (Microsoft) was used for data storage, and Prism 5 (GraphPad, Inc.) was used for all statistical analysis. One-way repeated-measures analysis of variance (ANOVA) with Bonferroni’s multiple-comparison correction was used to compare doubling times, and nonlinear transformation was used to calculate the LD_50_ of hydroxyurea. Linear regression was used to compare the slopes of time-dependent parasitemia curves, and log rank (Mantel-Cox) analysis was applied to survival curves. All data are mean values ± SEM. *P* values of ≤0.05 were considered significant.
